# Synthesis, crystal structure, vibrational study, optical characterization, Hirshfeld surface analysis and dielectric studies of a new indium-based hybrid material formulated as [(C_9_H_8_N)_2_(InCl_6_)·2(H_2_O)]†

**DOI:** 10.1039/d5ra01127b

**Published:** 2025-04-28

**Authors:** Hajer Khachroum, Abdallah Ben Rhaiem, Mohammed S. M. Abdelbaky, Mohamed Dammak, Santiago García-Granda

**Affiliations:** a Laboratory Inorganic Chemistry, Faculty of Sciences of Sfax, University of Sfax Sfax 3000 Tunisia khachroum.hajer2015@gmail.com; b Departamento de Química Física y Analítica, Universidad de Oviedo-CINN Oviedo 33006 Spain; c Departamento de Química Física, Facultad de Ciencias Químicas, Universidad de Salamanca Salamanca E-37008 Spain; d Laboratory LaSCOM, Faculty of Sciences of Sfax, University of Sfax BP1171 Sfax 3000 Tunisia

## Abstract

A newly developed indium-based hybrid compound, [(C_9_H_8_N)_2_(InCl_6_)·2(H_2_O)], was successfully synthesized using a slow evaporation method at room temperature. Scanning electron microscopy (SEM) and energy-dispersive X-ray spectroscopy (EDX) were employed to observe the morphology and chemical composition of the particles. Structural analysis was performed through crystal X-ray diffraction (SXRD) and powder X-ray diffraction (PXRD) and revealed that the studied material crystallized in the triclinic *P*1 space group. The atom packing in this structure was characterized by the presence of alternating organic and inorganic layers along the *b*-axis. These arrangements were stabilized through multiple hydrogen bonds and centroid–centroid stacking interactions occurring between nearly parallel organic cations. Vibrational and optical properties were also explored using FT-IR and UV–Vis methods, respectively. Additionally, thermal analysis was performed *via* TGA/DTA and DSC measurements to assess the thermal stability and phase transformation of the title compound. Analysis of the Hirshfeld surface was carried out to examine the interactions between molecules. This allowed a quantitative assessment of the relative contribution of these interactions in the crystal structure. AC conductivity measurements (10^−6^ Ω^−1^ cm^−1^) confirmed the semiconductor character of the compound. The conductivity mechanism was attributed to the correlated barrier hopping (CBH) mechanism. Furthermore, electrical modulus measurements demonstrated the presence of grain effects.

## Abstract 2

The AC conductivity response of the [(C_9_H_8_N)_2_(InCl_6_)·2(H_2_O)] compound aligns with the Jonscher power law at different temperatures. The decrease in *S* indicates that the transport mechanism in this material is governed by the CBH mechanism.

## Graphical abstract

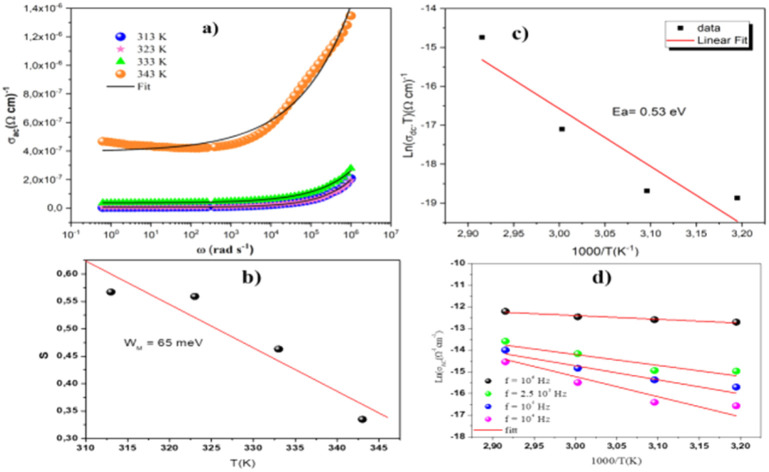

## 1. Introduction

In recent years, there have been significant advancements in organic–inorganic hybrid materials owing to their distinct structures combining inorganic frameworks with organic molecules. This combination typically occurs at the molecular level and results in properties that are distinct from those of the individual components.^1^ Because of their hybrid structure, these materials exhibit a wide range of properties, including unusual topological characteristics and superior magnetic, luminescence, catalytic, electrical, dielectric and ferroelectric properties.^2–11^ As per Sanchez's lecture on hybridization, the interaction between the organic and inorganic components in such hybrids is limited to weak bonds, such as hydrogen bonds, van der Waals interaction, or ionic interaction. Such materials are incredibly intriguing as they provide the foundation for productive applications across numerous industries, such as optical sensors, signal processors, organic light-emitting diodes,^12^ electrical conductivity, photochemistry, medicine and photocatalysis.^13–21^ Herein, as a member of the group (III) family, indium was selected for the study as it is a heavy metal that finds extensive utilization in various applications, ranging from industrial sectors^22^ to semiconductors,^23^ gas sensors, and solar cells.^24^ Therefore, organic–inorganic hybrid materials based on indium, such as those with the formula A_*n*−3_[In^III^X_*n*_] (A = organic ammonium cations; when X = Cl or Br, *n* = 4, 5, or 6; when X = I, *n* = 4), have attracted attention for their structural diversity and electrical characteristics. In-based organic–inorganic hybrid molecules offer facile synthesis and structural flexibility. Hence, a variety of In^3+^ compounds have been reported.^25–28^

Inspired by such studies, we present the synthesis, structure, spectroscopic measurements, optical properties, thermal analysis, and Hirshfeld analysis of a novel [(C_9_H_8_N)_2_(InCl_6_)·2(H_2_O)] hybrid compound.

## 2. Synthesis

An aqueous solution of InCl_3_·6H_2_O (99.99% purity, Sigma-Aldrich) was mixed with a solution of quinoline (C_9_H_7_N, ≥99% purity, Sigma-Aldrich) in ethanol (≥99.8% purity, Sigma-Aldrich) in an equimolar ratio. The reaction proceeded with a precise stoichiometric ratio, as shown below:2(C_9_H_7_N) + InCl_3_·6H_2_O + 6HCl → [(C_9_H_8_N)_2_(InCl_6_)·2(H_2_O)].

After stirring for 30 min, the mixture was left to dry at room temperature. Within a few days, well-defined brown single crystals were obtained, which were suitable for X-ray diffraction analysis and further studies.

## 3. X-ray crystallography

A glass fiber with tiny crystals of the new compound attached to it was affixed to an Agilent Gemini CCD diffractometer. Intensity data sets were obtained using Mo-Kα (*λ* = 0.071073 Å) radiation at room temperature. Data were collected, and the structures were solved using a direct method through SHELEXS-97.^29^ Based on F2, structural refinements were carried out using SHELXLE^30^ with the Olex-21.5 alpha program.^31^ All non-hydrogen atoms were refined anisotropically. Most hydrogen atom positions were calculated geometrically. The diagrams of the crystal structures and asymmetric units were created using Mercury 3.8.^32^ Table S1† presents partial atomic positions and similar isotropic thermal parameters, while anisotropic displacement parameters are listed in Table S2.† Furthermore, the selected bond lengths (Å) and angles (°) are given in Table S3.† Additionally, in order to identify the morphology and elemental content of the title compound, scanning electron microscopy (SEM) coupled with energy-dispersive X-ray (EDX) analysis was performed using a QUANTA microscope. The phase composition of the sample was verified using a Siemens D5000 powder diffractometer with Cu-Kα radiation (*α* = 1.54056 Å) at room temperature and an angle range of 5°–50°. Using the Crystal Explorer software version 21.5, one can determine intermolecular interaction within a crystal structure by utilizing the calculated Hirshfeld surface. This method allows specific molecules to be analyzed in the entirety of the crystal.

### 3.1. Infrared spectroscopy and UV–Vis measurements

Data were gathered using a PerkinElmer FT-IR spectrometer employing KBr pellets in the spectral range of 4000–400 cm^−1^. The measurement of UV–Vis absorption was conducted at room temperature using a PerkinElmer Lambda 35 UV/Vis spectrophotometer over the wavelength range of 200–800 nm.

### 3.2. Thermal study

A SETARAM DSC 131 ks device (featuring Pt containers and Al_2_O_3_ as the reference) was used to collect differential scanning calorimetry (DSC) readings of the raw powder. The temperature was increased from room temperature to 800 K at a steady rate of 5 K min^−1^ in a constant stream oxygen atmosphere using 3.121 mg of the sample.

A TGA Q500 TA device was employed to analyze the powdered samples (20.428 mg enclosed in an alumina crucible) over a temperature range from 300 to 1000 K in an O_2_ atmosphere. We maintained a heating rate of 10 K min^−1^ to ensure accuracy of the results.

### 3.3. Electrical measurements

Sample pellets 8 mm in diameter and 1 mm in thickness were fabricated and thoroughly analyzed to determine the sample's electric properties. The data were collected using a Solartron 1260 analyzer to perform complex impedance measurements. The temperature range tested was 313 to 353 K, while the frequencies ranged from 0.1 to 10^7^ Hz. To improve precision, the big faces of the pellet were coated with silver.

## 4. Results and discussion

### 4.1. SEM-EDX analysis

**Fig. 1:**
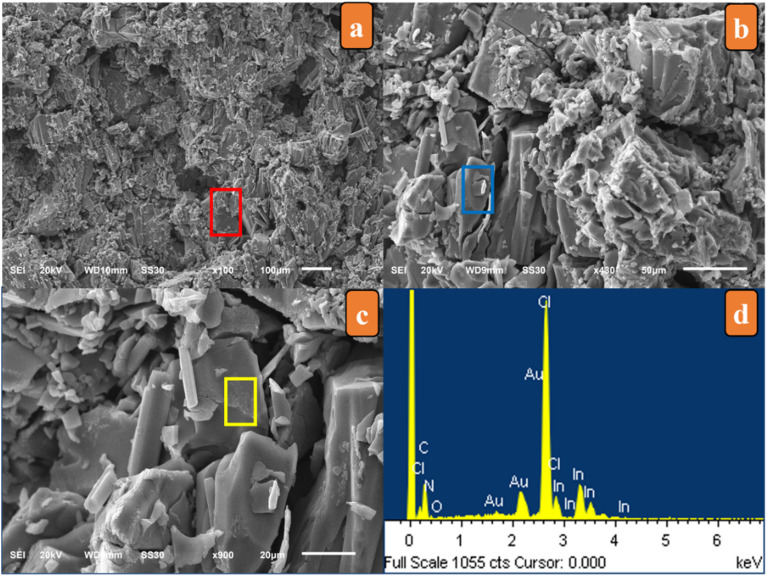
SEM images and EDX spectrum of [(C_9_H_8_N)_2_(InCl_6_)·2(H_2_O)] recorded at room temperature.

SEM micrographs displayed the crystal fragments with a homogeneous distribution and flat surface, indicating their good crystal quality (Fig. 1). EDX measurements of the sample were conducted, and indicated the presence of In, Cl, N, C and O, which were non-hydrogen constituent elements.

### 4.2. PXRD analysis

**Fig. 2:**
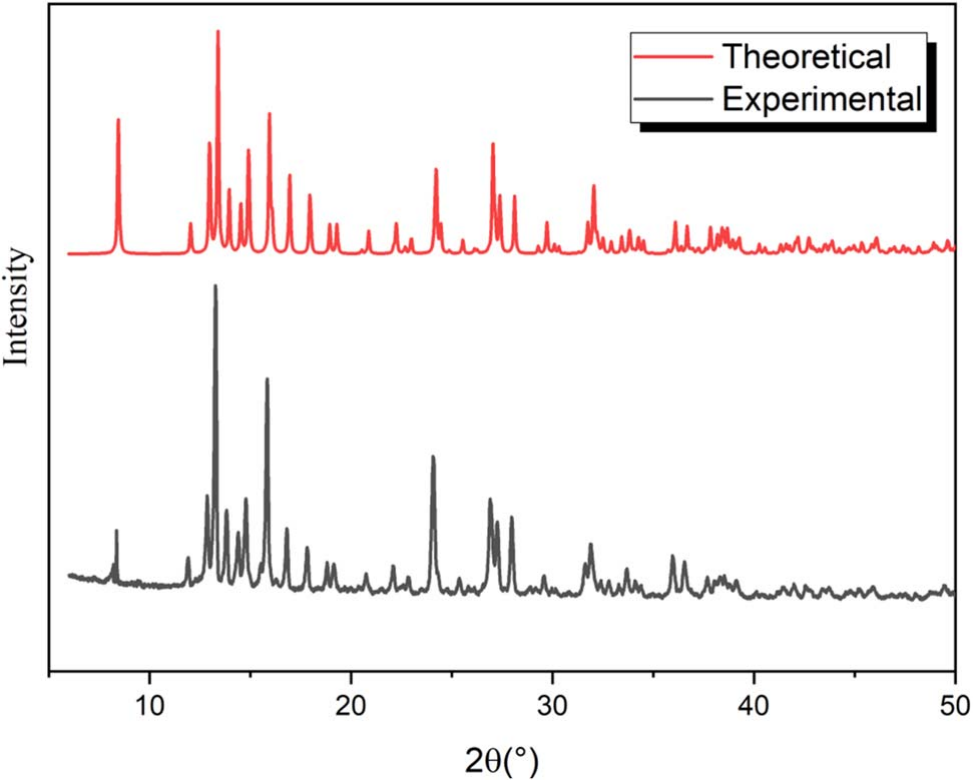
Comparison of the theoretical and experimental diffractograms of [(C_9_H_8_N)_2_(InCl_6_)·2(H_2_O)] at room temperature.

Comparison of the measured PXRD pattern and that simulated from single-crystal data verified a single phase without any visible impurities, confirming the remarkable purity of the title compound bulk crystal, as shown in Fig. 2.

### 4.3. Structure description

The title compound crystallized at room temperature was classified as an organic–inorganic hybrid, and its structure adopted the centrosymmetric *P*1̄ space group. Selected crystallographic parameters and experimental conditions are listed in Table 1.

**Table 1:** Main crystallographic characteristics and refinement parameters of [(C_9_H_8_N)_2_(InCl_6_)·2(H_2_O)]

Formula	[(C_9_H_8_N)_2_(InCl_6_)·2(H_2_O)]
Formula weight	623.88
Temperature/K	298.15
Crystal system	Triclinic
Space group	*P*1̄
*a*/Å	7.4718(3)
*b*/Å	8.1648(5)
*c*/Å	10.7809(5)
*α*/°	76.573(4)
*β*/°	80.404(4)
*γ*/°	66.171(5)
Volume/Å^3^	583.22(6)
Z	1
*ρ*_calc_/g cm^−3^	1.776
*μ*/mm^−1^	1.718
*F*(000)	309.0
Crystal size/mm^3^	0.316 × 0.044 × 0.043
Data collection instrument	Kappa CCD
Radiation	Mo Kα (*λ* = 0.71073)
2*θ* range for data collection/°	5.548 to 62.928
Index ranges	−10 ≤ *h* ≤ 10
	−11 ≤ *k* ≤ 11
	−15 ≤ *l* ≤ 15
Reflections collected	16 579
Independent reflections	3625
Goodness-of-fit on *F*^2^	1.050
Final *R* indexes	*R*_1_ = 0.07, wR_2_ = 0.18
Largest diff. peak/hole/e Å^−3^	0.75/−1.84
CCDC deposition number	2323035

**Fig. 3:**
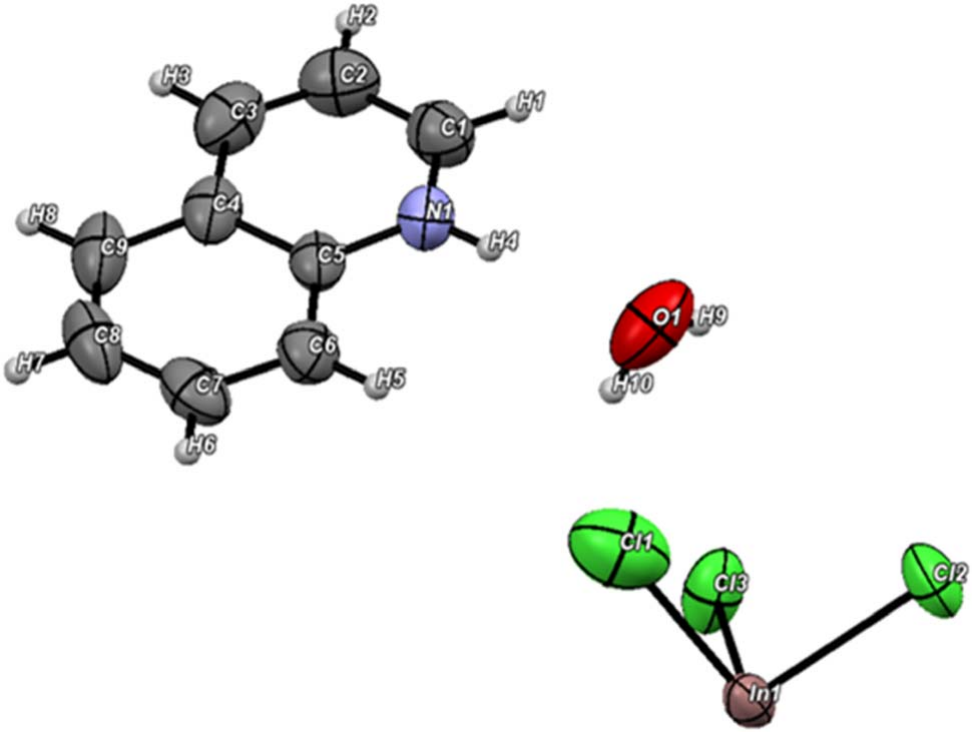
Asymmetric unit of [(C_9_H_8_N)_2_(InCl_6_)·2(H_2_O)] with thermal ellipsoids (50%).

The unit cell parameters are as follows: *a* = 7.4718(3) Å, *b* = 8.1648(5) Å, *c* = 10.7809(5) Å, *α* = 76.573(4)°, *β* = 80.404(4)°, *γ* = 66.171(5)°, V = 583.22(6) Å^3^, and *Z* = 1. As shown in Fig. 3, the asymmetric unit of the investigated compound consists of one crystallographically protonated quinolinium (C_9_H_8_N)^+^ cation, a half [InCl_6_]^2−^ and one uncoordinated water molecule.

**Fig. 4:**
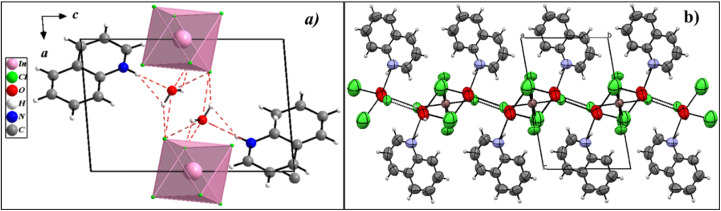
(a) Projection of the structure in the (b and c) plane. (b) Packing structural arrangements of [(C_9_H_8_N)_2_(InCl_6_)·2(H_2_O)] along the *b*-axis showing hydrogen bonds.

Hence, upon scrutinizing the atomic configuration present in the crystalline framework of the investigated compound, it could be inferred that the inorganic units were interspersed with organic chains in a sequential manner along the crystallographic *b*-axis and linked together by O–H⋯Cl hydrogen bonds from the free water molecules situated between the two layers. On the other hand, the packing diagram of the crystal structure along the *b*-axis illustrated an alternating arrangement of inorganic layers, positioned at *x* = ½*c*, and organic chains, extending along the crystallographic *a*-axis at *x* = 0 and *x* = 1. Interspersed between these two layers, water molecules contribute to the overall structural stability by reinforcing cohesion through hydrogen bonding and other intermolecular interactions, as projected in Fig. 4(a) and (b).

The inorganic part consisted of an octahedral [InCl_6_]^2−^ unit with In–Cl distances between 2.455(16) and 2.521(3) Å and Cl–In–Cl angles ranging from 87.76(8)° to 92.24(8)°, forming a slightly distorted octahedron.^33–36^ Cl⋯Cl interactions provide the necessary cohesion between the [InCl_6_]^2−^ anions in the inorganic column. The closest distance between chlorine atoms of the nearest [InCl_6_]^2−^ entities was 4.225 Å. This weak halogen–halogen contact is sufficient to ensure the cohesion of the inorganic columns and is known to generate weak antiferromagnetic interactions. Regarding the organic component, it consisted of two separate quinolinium organic cations that were protonated. The C–C and C–N distances within this component ranged from 1.337(11) to 1.417(10) Å and from 1.304(9) to 1.368(8) Å, respectively. Four categories of hydrogen bonds are responsible for maintaining the stability and unity of the structure. Furthermore, water molecules play a supplementary role in stabilizing the structure. This is achieved through the N–H⋯O bond and the hydrogen *via* C–H⋯Cl and O–H⋯Cl interactions, which provide a link between the cationic (C_9_H_8_N)^+^ entities and [InCl_6_]^2−^ anions. Each cationic group forms two hydrogen connections with two distinct clusters of inorganic moieties, creating a pseudo 3D hybrid class I structure. The hydrogen bonding information can be found in Table 2.

**Table 2:** Hydrogen bonds of [(C_9_H_8_N)_2_(InCl_6_)·2(H_2_O)]^a^

D–H⋯A	D–H (Å)	H⋯A (Å)	D⋯A (Å)	D–H⋯A (°)
O1–H10⋯Cl1	0.85	2.352(1)	2.909(1)	123.54(1)
N1–H4⋯O1	0.85	1.993(1)	2.815(1)	159.53(1)
O1–H9⋯Cl3 (i)	0.85	2.537(2)	3.229(2)	139.28(2)
C1–H1⋯Cl3 (i)	0.85	2.822(2)	3.595(2)	141.23(2)
C6–H5⋯Cl2 (ii)	0.93	2.998(3)	3.456(3)	112.10(3)
C1–H1⋯Cl1 (iii)	0.93	2.879(4)	3.489(4) (3)	124.28(4)
C2–H2⋯Cl1 (iii)	0.93	2.997(4)	3.462(4)	126.43(4)
C7–H6⋯Cl2 (iv)	0.93	2.914(5)	3.635(5)	135.31(5)
C9–H8⋯Cl3 (v)	0.93	2.709(6)	3.509(6)	144.66(6)
C3–H3⋯Cl2 (vi)	0.93	2.82(7)	3.69(7)	155.50(7)

a Symmetry codes: (i): −*x* + 1, −*y* + 1, −*z* + 1; (ii): −*x* + 1, −*y*, −*z* + 1; (iii): *x* − 1, *y* + 1, *z*; (iv): *x*, *y*, *z* − 1; (v): *x* + 1, *y*, *z* − 1; (vi): *x* − 1, *y* + 1, *z* − 1.

### 4.4. IR spectroscopy analysis

In order to obtain information on the crystal structure and determine the functional groups present, FT-IR spectroscopy was used. Our attention was primarily directed towards analysing the vibrations of the organic cations and water molecules. The process was conducted professionally to ensure accurate results and comprehensive analysis.

**Fig. 5:**
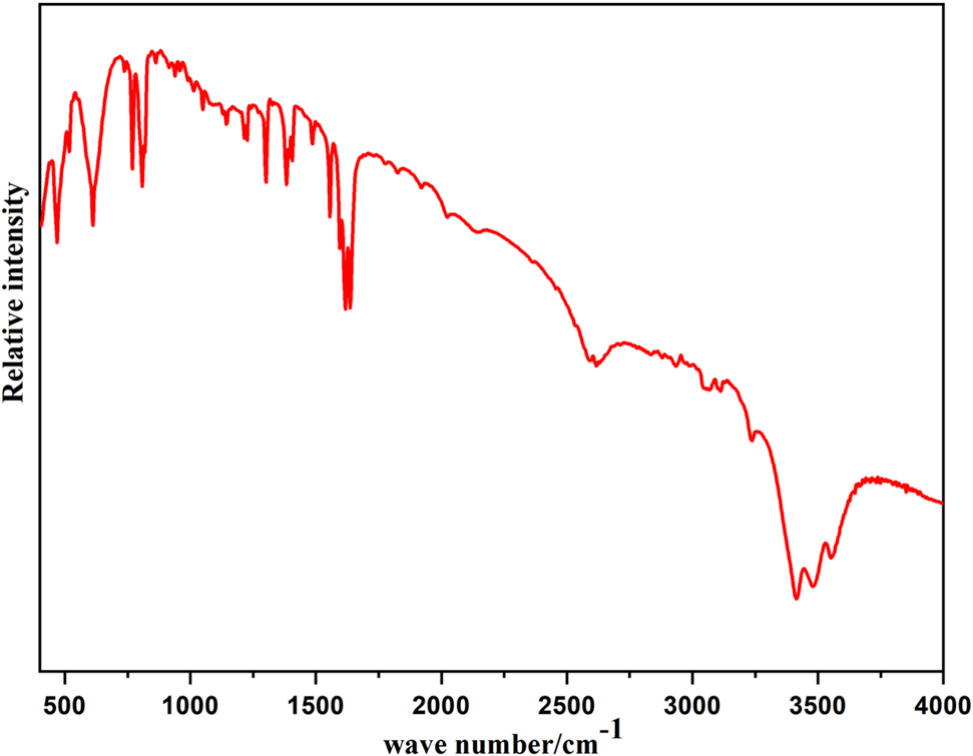
FT-IR spectrum of [(C_9_H_8_N)_2_(InCl_6_)·2(H_2_O)] at room temperature.

The recorded IR spectrum is shown in Fig. 5. The bands observed at 3558 and 3481 cm^−1^ were attributed to *υ*(O–H) vibrations.^37^ Meanwhile, the band observed at 1596 cm^−1^ was responsible for the (O–H) deformation vibration of water molecules, while the band located at 3417 cm^−1^ was related to the *υ*(N–H) vibrations of protonated quinoline. The bands located at 3051, 1051, 804, and 765 cm^−1^ were related to the *υ*(C–H) and *δ*(=C–H) of the quinoline ring. Furthermore, the (C=C) stretching band of the aromatic pyridine ring was displayed at 1568 cm^−1^. The bands observed at 990 and 1614 cm^−1^ were attributed to the (C–H) bending deformation vibration and (C=N) stretching mode of the pyridine ring. Also, the bands at 1620, 1555, and 1506 cm^−1^ could be recognized as *υ*(C–N) and *υ*(C–C) of the quinoline ring, as observed with other similar hybrid compounds.^20,35–40^

### 4.5. UV–visible absorption properties and Urbach energy

**Fig. 6:**
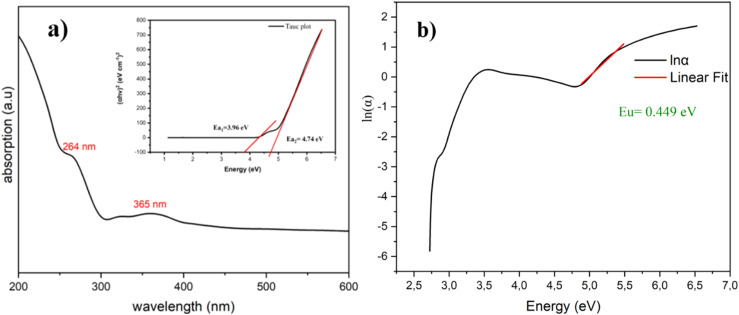
(a) UV–visible spectrum of [(C_9_H_8_N)_2_(InCl_6_)·2(H_2_O)]; inset shows the Tauc plot model. (b) Variation in ln(*α*) as a function of energy for [(C_9_H_8_N)_2_(InCl_6_)·2(H_2_O)].

The UV–visible absorption spectrum of the title compound was investigated in the range of 200–800 nm at room temperature (Fig. 6), and it exhibited two absorption bands located at 264 (3.96 eV) and 365 nm (4.74 eV). These two activation energies confirmed the semiconductor character of this compound. The band at 264 nm corresponded to the π–π* and n–π* electron transitions of the coordinated ligand,^41^ while the peak at 365 nm was attributed to excitation from the inorganic layers in [InCl_6_]^2−^. This observation was in agreement with previous research on organic–inorganic hybrid materials.^35,42^ Under excitation, electrons transit from the valence band (VB) to the conduction band (CB), creating a hole in the valence band (VB), and emission occurs when the electron and hole recombine.

Next, Urbach tail analysis was conducted to gain an insight into the material's disorganisation. In this situation, Urbach's rule illustrates how the absorption edge broadens and a band tail forms. The Urbach tail energy (*E*_U_) is used to assess the width of the tails caused by localised states at the absorption edge.^43^ The Eu value can be found using the following equation:ln(*α*) = ln(*α*_0_) + *hν*/*E*_u_,where *α*_0_ is a constant, *E*_U_ represents the Urbach energy (eV), and *hν* is the photon energy (eV). As illustrated in Fig. 6(b), the tail did not fit a single slope accurately in the ln(*α*) *versus* photon energy plot. The investigated compound exhibited a specific *E*_U_ value (*E*_U_ = 0.449 eV) that was comparable to other band tails reported in some indium-based hybrid perovskites, such as (CsAgNaInBiCl).^44^ Notably, that compound exhibited a higher *E*_U_ value compared to our hybrid material, potentially due to a higher defect density within the cell.^45^ Consequently, the recombination rate in perovskite compounds increases as the *E*_U_ rises, likely resulting from the higher density of localized states within the band gaps.

### 4.6. Hirshfeld surface analysis

**Fig. 7:**
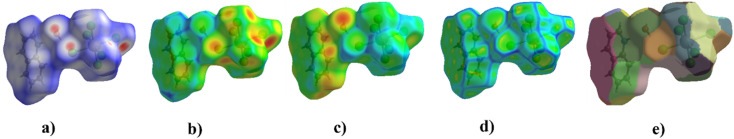
Hirshfeld surface analysis of [(C_9_H_8_N)_2_(InCl_6_)·2(H_2_O)]: (a) *d*_norm_, (b) *d*_i_, (c) *d*_e_, (d) shape index, (e) curvedness, and (f) fragment patch.

To gain a thorough understanding of the interactions between molecules within the crystal structures, we conducted analyses utilizing Hirshfeld surfaces calculated through Crystal Explorer 21.5.^46^ Electron distribution, which is calculated by adding the electron densities of spherical atoms, serves as the foundation for constructing the crystal structure.^47^ The Hirshfeld surface produced by a specific crystal structure and a set of spherical atomic electron densities is singular.^48^ The Hirshfeld surface is composed of points where the molecule of interest's contribution to the electron density is equivalent to the contribution made by all other molecules.^49^ Two distances, *d*_e_ and *d*_i_, are established for each point on the iso-surface. The Hirshfeld surface distance to the closest atom outside the surface is represented by the symbol *d*_e_. The value of *d*_i_ represents the separation between the closest atom inside the surface and the Hirshfeld surface. The standardised contact distance, or *d*_norm_, is determined by taking into account the atom's vdW and *d*_e_ radii using the following equation:*d*_norm_ = (*d*_i_ − *r*^vdW^_i_)/*r*^vdW^_i_ + (*d*_e_ − *r*^vdW^_e_)/*r*^vdW^_e_,where *r*^vdW^_i_ and *r*^vdW^_e_ are the van der Waals radii of the appropriate atoms internal or external to the surface. Red, blue, and white are the colours used to graphically represent the value of *d*_norm_, which can be positive or negative based on the intermolecular interactions. The Hirshfeld surface of the asymmetric unit, mapped with the *d*_norm_ property, highlights large circular depressions characterized by a deep red color, as shown in Fig. 7(a). These depressions serve as indicators of hydrogen bonding contacts, which can be attributed to various interactions, such as N–H⋯Cl, O–H⋯Cl and C–H⋯Cl interactions. Furthermore, the presence of white patches on the surface was attributed to H⋯H van der Waals contacts. The ideal measures for detecting π–π interactions, namely, the curvedness and the shape index, are depicted in Fig. 7(d) and (e). For our present compound, the curvedness maps showcase vast flat green areas, emphasized by a bold blue outline encircling the pyridinium cycle.

**Fig. 8:**
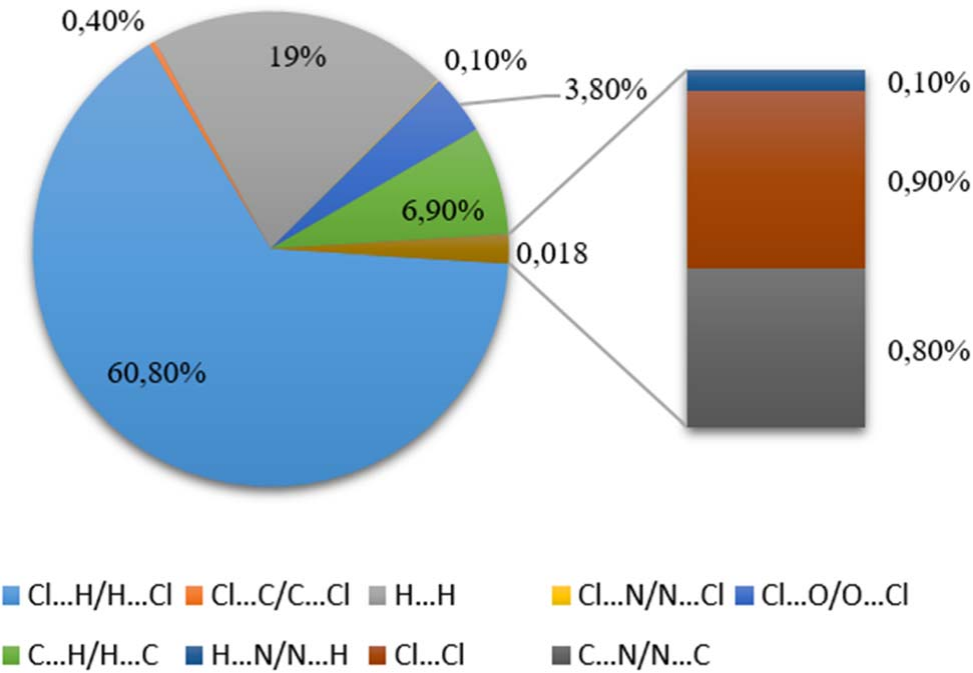
2D fingerprint plots of the full intermolecular interactions and histogram showing the percentage of contacts.

Fig. 8 presents the 2D fingerprint plot using the standard view with the graph's axes displaying the distance scales of de and di obtained through Hirshfeld surface analysis. This graph depicts a summary of the intermolecular contacts in the crystal. A major emphasis here is placed on the H⋯Cl/Cl⋯H intermolecular interactions, which notably contribute to 60.8% of the Hirshfeld surface. Further it is essential to highlight that H⋯H interactions also make a significant contribution to the total Hirshfeld surfaces of the crystal structure, constituting up to 19%. By dissecting the 2D fingerprint plots that highlight the nearby contacts, it becomes possible to reveal and analyze each contribution from the various interactions obtained from the complete fingerprint. The C⋯H interactions can be considered as the third noticeable interactions, accounting for up to 6.90%. Additionally, the C⋯C contacts between the organic cations are in the range of 6% of the total close contacts, which suggests the presence of π–π interactions between the involved atoms. In addition to these interactions, there are also red hollows on the second side of the fingerprint plot, such as N⋯H (1.8 and 1.1%) and N⋯C (0.8%), of the Hirshfeld surface.

Succinctly and based on the quantitative analysis, we could evidently conclude that the predominant contributors to the overall surface are the H⋯Cl/Cl⋯H interactions and the closely packed H⋯H contacts. These interactions serve as the primary driving force within the crystal structure of the new studied hybrid compound.

### 4.7. Thermal decomposition

Next, the thermal stability and breakdown characteristics of the synthesized complex were investigated using DTG-TG/DTA and DSC analyses. These factors hold particular significance in electronic device manufacturing, where a crystal's ability to endure laser light exposure and yield precise results is crucial. We conducted thermal analysis on finely crushed powder samples.

**Fig. 9:**
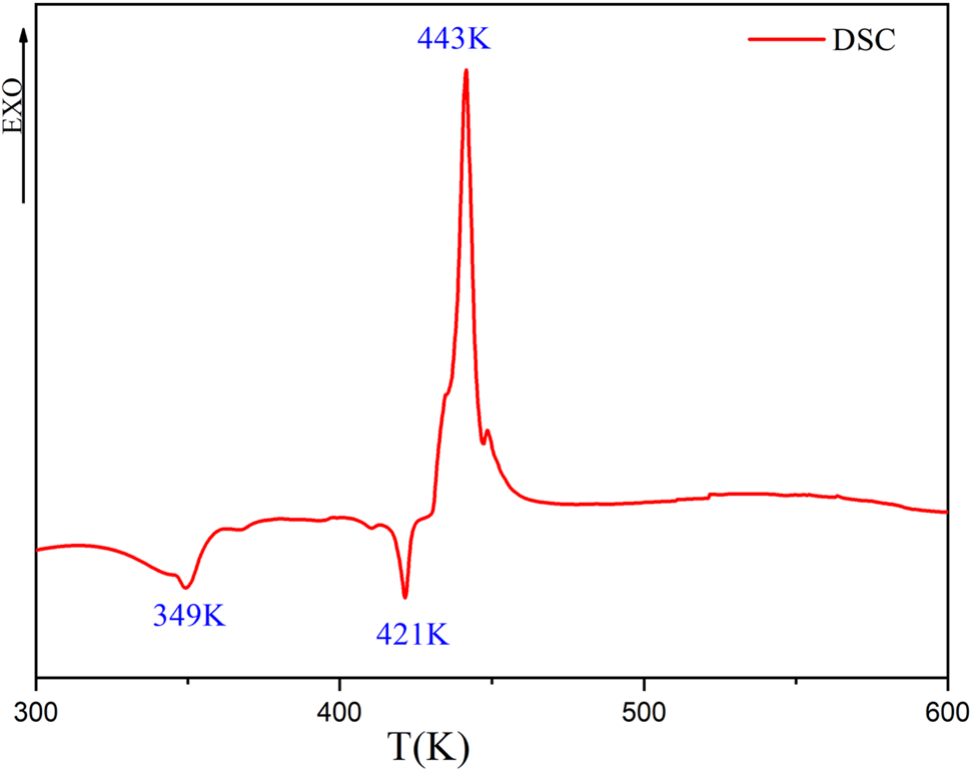
DSC curve of [(C_9_H_8_N)_2_(InCl_6_)·2(H_2_O)] recorded at a heating rate of 10 K min^−1^ in an oxygen atmosphere.

The DSC heating curve was recorded in the temperature range of 300–600 K using 3.12 mg of the sample at a heating rate of 10 K min^−1^, and the result is displayed in Fig. 9.

**Fig. 10:**
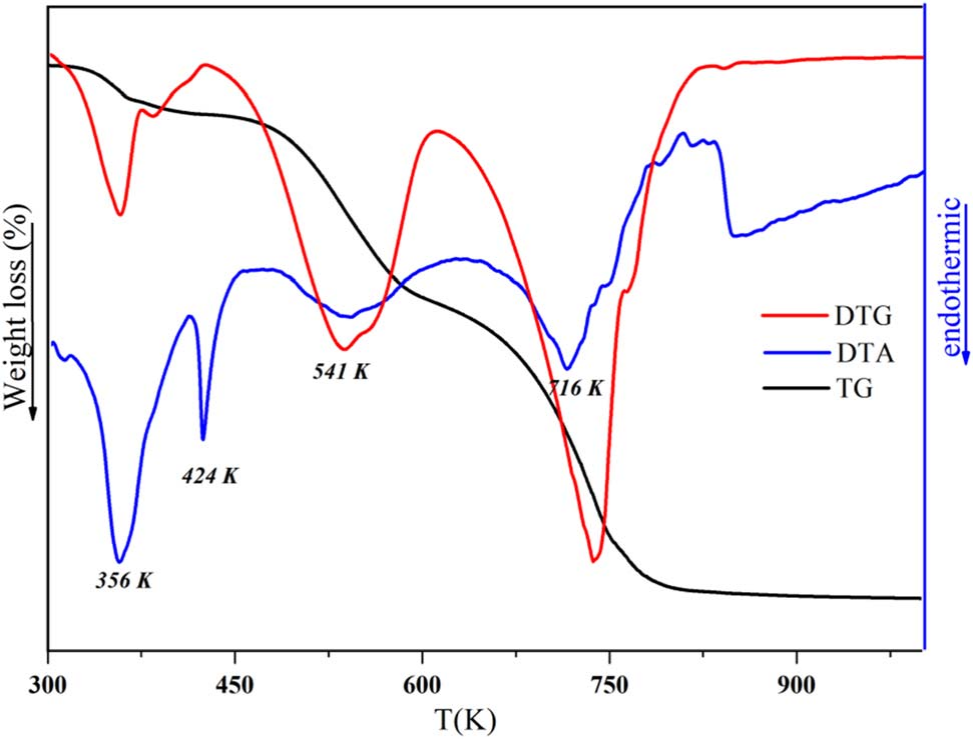
DTG-TG/DTA curves of [(C_9_H_8_N)_2_(InCl_6_)·2(H_2_O)] from RT to 1200 K.

In fact, three peaks were observed as the temperature increased. The first endothermic peak was detected at 349 K (Δ*H* = −159.18 J g^−1^, *E*_a_ = 65.40 kJ mol^−1^) and was assigned to the release of two water molecules. The second endothermic peak in the heating process around 421 K observed in the DSC corresponded to the fusion, with an enthalpy (Δ*H*) of −52.95 J g^−1^ and an activation energy (*E*_a_) of 78.87 kJ mol^−1^. The third exothermic peak detected at 443 K (Δ*H* = 524.81 J g^−1^ and *E*_a_ = 82.94 kJ mol^−1^) corresponded to the decomposition of the material. The thermal stability of the crystal was examined using thermogravimetric and differential thermal analysis techniques (Fig. 10). The results obtained by these methods were in agreement with each other. The TG curve showed that the substance was stable until it reached 308 K, and, then, it decayed, losing 92.34% of its total initial weight. However, the first loss of mass was related to the dehydration of the two lattices of water (1st loss of mass = 2H_2_O), which occurred between 308 to 361 K, losing 5.94% of the total initial weight (calculated at 5.77%), which is comparable with other reported hybrid compounds.^50–52^ This loss was associated with two endothermic peaks in the DTA and DTG curves at around 356 K. The fine and intense dip observed in DTA at 424 K indicated the melting point of the substance. It could obviously be noted that the sample decomposed immediately after melting in two stages, which was further verified by the DTA and DTG curves. The second degradation was related to the elimination of the two organic cations (2nd loss of mass = 2(C_9_H_8_N)^+^), which began at 361 K and proceeded until 631 K, and, in this process, 36.54% (calculated at 41.67%) of the total initial mass was lost. This loss of mass was accompanied with an endothermic peak in DTG and DTA at 541 K. Additionally, the third phase of the process happened between 600 and 750 K and involved the pyrolysis of the inorganic anion (3rd loss of mass = 6(HCl)), which further contributed to a mass loss of 49.86% (52.54% calculated). This degradation was accompanied by two endothermic peaks in both DTA and DTG at 716 K. Finally, 7.655% of the total mass was obtained as a result of the decomposition, which may be related to the formation of In_2_O_3_ residue.

### 4.8. Nyquist diagram

**Fig. 11:**
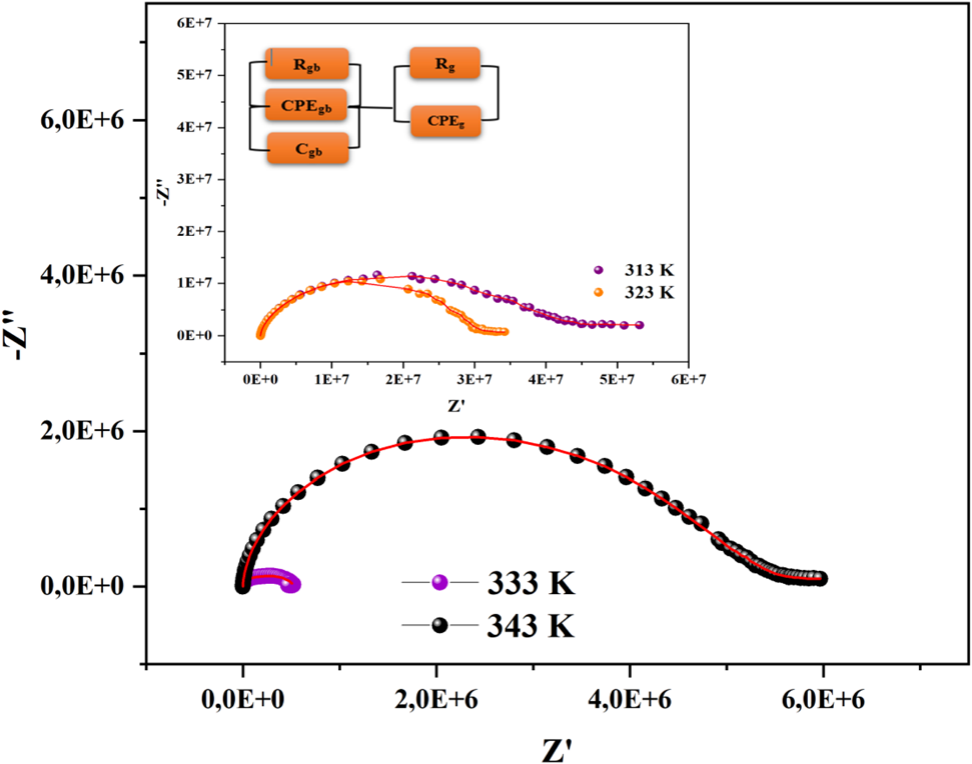
Nyquist diagrams of the [(C_9_H_8_N)_2_(InCl_6_)·2(H_2_O)] sample.

One effective method for characterizing the electrical behavior of a sample is impedance spectroscopy. This method works well for separating the effects of electrode polarization, grain boundaries, and grain contribution. An appropriate equivalent circuit must be proposed in order to demonstrate the material's electrical characteristics and comprehend the response that is received. The Nyquist plots (−*Z*′′ *vs. Z*′) of [(C_9_H_8_N)_2_(InCl_6_)·2(H_2_O)] at temperatures ranging from 313 K to 343 K are shown in Fig. 11.

An analogous circuit with two cells representing the grains and the grain boundary in this material yielded the best fit. The first cell consisted of a parallel combination of the resistance (*R*_gb_), capacitance (*C*_gb_) and a constant phase element (CPE_gb_). The second consisted of a parallel combination of the resistance (*R*_g_) and a constant phase element (CPE_g_). Undoubtedly, the existence of a constant phase element (CPE) in these circuit models accounts for the semicircles' observed depression as well as their non-Debye behavior.^27^ The impedance of the CPE contribution was computed using the type of empirical function described below:
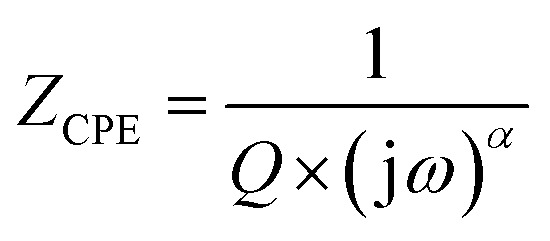


In the Nyquist notation, the exponential *α* determines the angle of phase (*β*), *β* = (1 − *α*)π/2, between the *Z*′ axis and the radius of the semicircle, while the proportional factor (*Q*) indicates the value of the capacitance of the CPE element. The parameter values obtained from the equivalent circuit utilized in the electrical response are grouped in Table 3. It is clear that as the temperature was increased, the compound's grain and grain border resistance values decreased, indicating that our sample behaved like a semiconductor.^53,54^ Furthermore, the low alpha values confirmed the significant interaction between the dipoles in this material.^55,56^

**Table 3:** Parameter values obtained from the equivalent circuit

Temperature	*R*_gb_ (10^6^ Ω)	*Q*_gb_ (10^−9^ F)	*α*gb	*C*_gb_ (10^−11^ F)	*R*_g_ (10^5^ Ω)	*Q* (10^−10^ F)	*α*_g_
40	47.19	1.645	0.451	3.50	1.64 × 10^5^	22.12	0.28
50	31.60	6.89	0.549	3.21	1.02 × 10^5^	11.15	0.88
60	5.640	2.576	0.438	3.53	9.23 × 10^4^	8.23	0.57
70	0.644	53.19	0.35	3.39	8.11 × 10^4^	7.67	0.52

### 4.9. AC electrical conductivity

**Fig. 12:**
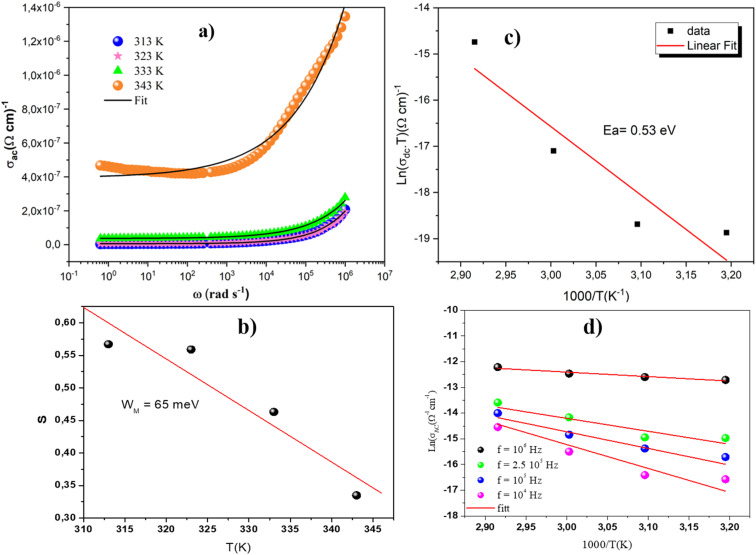
(a) Frequency dependence of AC conductivity at different temperatures, (b) linear fit of the exponent *S* as a function of temperature, (c) dependence of ln(*σ*_dc_·*T*) on temperature in [(C_9_H_8_N)_2_(InCl_6_)·2(H_2_O)], and (d) fitting of AC conductivity at different frequencies using the CBH model.

AC conductivity (*σ*_ac_) is a fundamental property that reflects the electrical dynamics of a sample, including its conductivity, capacitance, and the loss factor, while helping classify the conduction mechanism. It is determined from complex impedance measurements using the following formula:
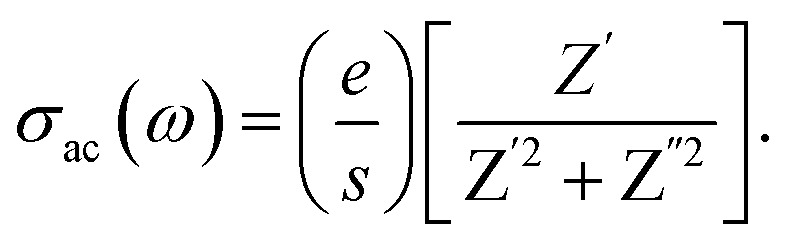


The AC conductivity variation for [(C_9_H_8_N)_2_(InCl_6_)·2(H_2_O)] across several fixed temperatures is presented in Fig. 12(a). This data indicate that the AC conductivity was nearly constant at lower frequencies but showed dispersion at higher frequencies, typical of *ω*^*s*^. The observed low *σ*_ac_ conductivity values, around 10^−6^ Ω^−1^ cm^−1^, confirmed its proton-conduction properties.^57,58^ At lower frequencies (10^−1^ < *ω* < 5 × 10^3^), the electrical current enabled the charge carriers to move over greater distances, which indicated direct current conductivity (*σ*_dc_).^59,60^ As a result, the principal motions of the charge carriers decreased with increasing frequency.^61^ As the frequency increased (*ω* > 5 × 10^3^), the leaping frequency of the charge carriers is improved, resulting in an accelerated conduction pathway and enhanced conductivity of the sample. On top of that, this feature denotes a thermally activated process beginning by a boost in the energy levels of the charge transports, which was dominated by the motion of the H^+^ protons due to breaking of the O–H⋯Cl, C–H⋯Cl and O–H⋯N bonds in the structure of the investigated material. The Jonscher's power law^62^ is commonly employed to analyze the phenomenon of conductivity dispersion, and is expressed as:*σ*_ac_(*ω*) = *σ*_dc_ + *Aω*^*s*^,where *σ*_dc_ is the direct current conductivity, *A* is a constant regulating the strength of the polarizability, *ω* = 2π*f* is the angular frequency, and *s* (≤1) denotes the degree of contact between mobile ions and the enclosing crystalline structure.

To better understand the primary AC conduction mechanism of the material, we investigated the temperature dependence of the exponent *s*, as shown in Fig. 12(b). It could be noticed that the values of *s* showed an inverse variation with temperature. The observed decrease in *s* values at higher temperatures suggests that the correlated barrier hopping (CBH) model could effectively describe the charge transport mechanism in this region, involving the hopping of charge carriers across potential barriers.^63^

Additionally, the fitted data of DC conductivity are shown in Fig. 12(c), and the dependence is represented by the Arrhenius equation:*σ*(*T*) = *A* exp(−*E*_a_/*k*_B_*T*),where *A* represents the pre-exponential factor, *k*_B_ is the Boltzmann constant and *E*_a_ is the thermal activation energy for ion migration. From the plotted graph, we can deduce that the dc conductivity curve boosted with increasing the temperature. Linear fitting the data yielded an activation energy value of 0.53 eV across the temperature range of 313–343 K. This observation indicates that a thermally triggered hopping process governed the conduction mechanism.

In the CBH model, the *S* exponent was evaluated using the following equation:
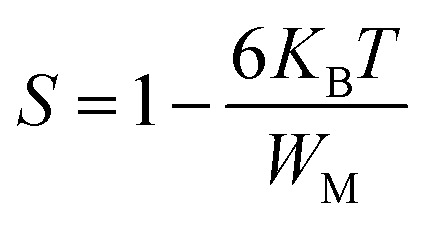


The linear fit of *S* as a function of temperature can be used to determine the value of the potential barrier *W*_M_, as mentioned in Fig. 12(b). We discovered that *W*_M_ = 65 meV, which was less than 25% of the activation energy (0.53 eV), demonstrating that a single polaron mediated this CBH model. The expression for AC conductivity for the single polaron correlated barrier jump model is as follows:
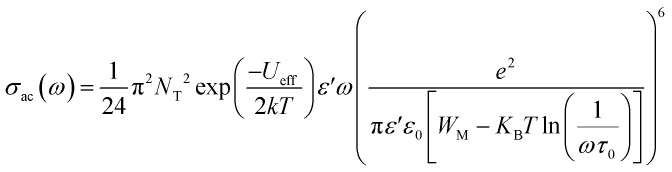
where *ε*_0_ is the vacuum permittivity, *ε*′ is the dielectric constant of the material, *N*_T_ is the number of state densities and *U*_eff_ is the effective potential energy. Our choice of conduction mechanism is supported by Fig. 12(d), which shows there was good agreement between the experimental and computed data of *σ*_ac_ (Table 4).

**Table 4:** Values of the parameters *N*_T_, *W*_m_ and *U*_eff_

*F* (Hz)	*N*_T_ (10^21^ eV m^−3^)	*W*_m_ (eV)	*U*_eff_ (eV)
10^6^	5.25	0.110	0.298
2.5 × 10^5^	4.81	0.079	0.850
10^5^	3.75	0.056	1.088
10^4^	2.88	0.028	1.57

Furthermore, it can be inferred that the conductivity of our compound (10^−6^ Ω^−1^ cm^−1^) was higher than that of the [C_4_H_12_N]_2_InCl_5_ hybrid compound (10^−7^ Ω^−1^ cm^−1^).^64^ This difference could be attributed to structural variations between the two materials.

### 4.10. Dielectric modulus

Analysis of electrical properties relies on a complex modulus formalism, which is well-suited for emphasizing the bulk response of the crystal sample and for uncovering phenomena such as electrode polarization and conductivity relaxation times.^65^

The calculation of the electric modulus (M*) follows the below equation:M* = j*ωC*_0_Z* = *M*′ + j*M*′′,where *M*′ and *M*′′ are the real and imaginary parts of the modulus, respectively, and *C*_0_ represents the vacuum capacitance.

**Fig. 13:**
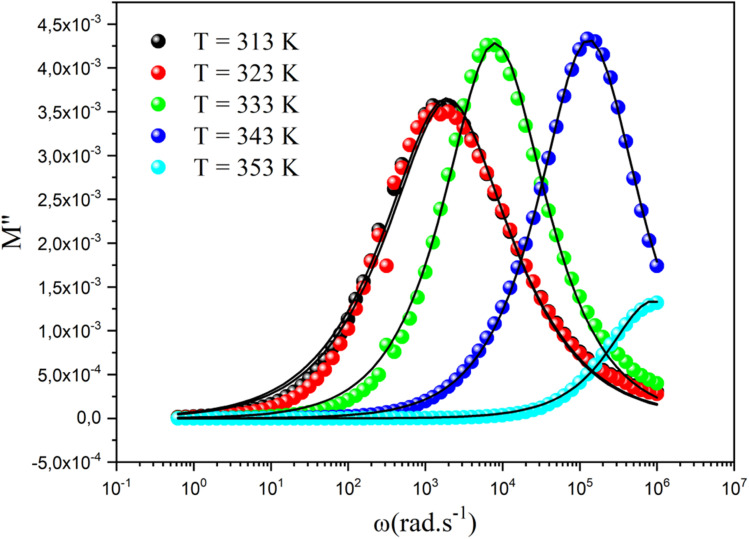
Frequency dependence of the imaginary part of the dielectric modulus at different temperatures.

The curve of the imaginary portion *M*′′ exhibited a distinctive asymmetric peak at all temperature points, revealing important characteristics about ion mobility, as presented in Fig. 13. As the temperature increased, these peaks were notably shifted toward higher frequencies, which serves as a clear indicator of the intricate association between the movements of the mobile charges carriers within the studied material.^66–68^ In addition, we can infer that this phenomenon was simulated by heat. The observed asymmetry in peak broadening was particularly significant, as it indicated a diverse distribution of relaxation times, each associated with different time constants. The frequency at which the modulus reached its maximum value 
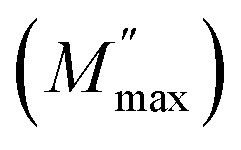
 at *ω*_max_ consistently shifted toward higher frequencies as the temperature rose, suggesting a hopping mechanism. This indicates that the imaginary part of the dielectric modulus was thermally activated. After the maximum frequency, the *M*′′(*ω*) values dropped with increasing frequency. In this plot, the existence of two relaxation domains could be discerned. The low-frequency region (*ω* < 10^3^ rad s^−1^) suggests that the charge carriers had the capability to traverse extended distances from one site to another through the material. Conversely, the peaks appearing in the high-frequency domain (10^3^ < *ω* < 10^6^ rad s^−1^) indicate that the charge transfers were confined within their potential wells, limiting their movement.

The overall nature and behavior of the modulus spectrum provides compelling evidence for the existence of a hopping mechanism, which plays a fundamental role in facilitating electrical conduction within the material's structure. The asymmetrical peaks in *M*′′ revealed a non-Debye behavior. The Kohlrausch–Williams–Watts (KWW) equation is used to examine the asymmetric pattern of the peaks in the imaginary part of the electric modulus:^69^
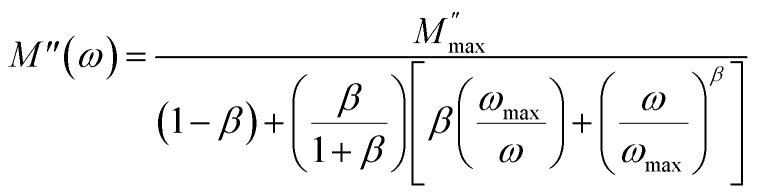


**Fig. 14:**
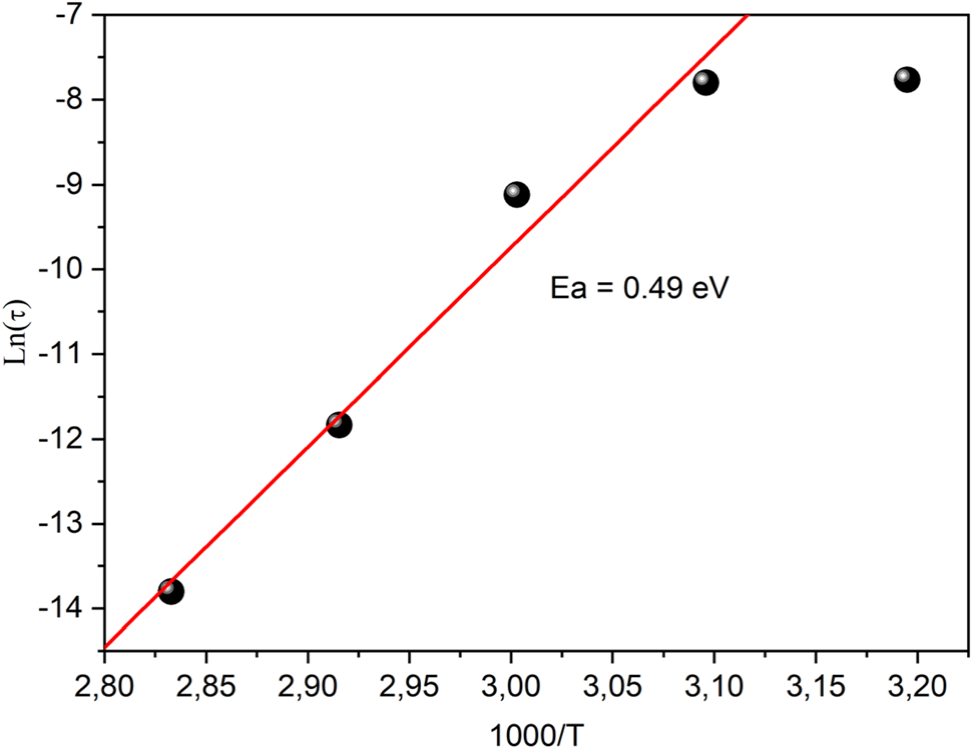
Variation in the relaxation time ln(*τ*) as a function of 1000/*T*.

The peak frequency (*ω*_max_), which corresponds to the alteration between long-range and short-range ionic mobility, allows us to determine the relaxation time (*τ*_m_). This critical transition is governed by the relationship *ω*_m_ × *τ*_m_ = 1, with *τ*_m_ denoting the ions' most probable relaxation time. From the fitted data of the dielectric modulus, we could extract the variation in ln(*τ*) as a function of 1000/*T*. This behavior followed the Arrhenius model and its associated activation energies, as shown in Fig. 14. From the slope of ln(*τ*) *versus* 1000/*T*, the activation energy was determined to be 0.49 eV, which coincides with the value derived from the slope analysis of ln(*σ*_dc_) *versus* 1000/*T*. This observation verified that the conduction mechanism was manifested by hopping transport.^63^

### 4.11. Dielectric permittivity

A dielectric material's polarizability is characterized by its relative permittivity (*ε*_r_) or dielectric constant (ε*), typically expressed as a complex value by the following equation:^70^ε*(*ω*) = *ε*′(*ω*) − i*ε*′′(*ω*)

The real component (*ε*′) quantifies the material capacity to store electrical energy, reflecting the alignment of dipoles within the dielectric. In contrast, the imaginary component (*ε*′′) represents energy dissipation due to frictional forces, which hinder charge displacements from staying in phase with the field variations.

**Fig. 15:**
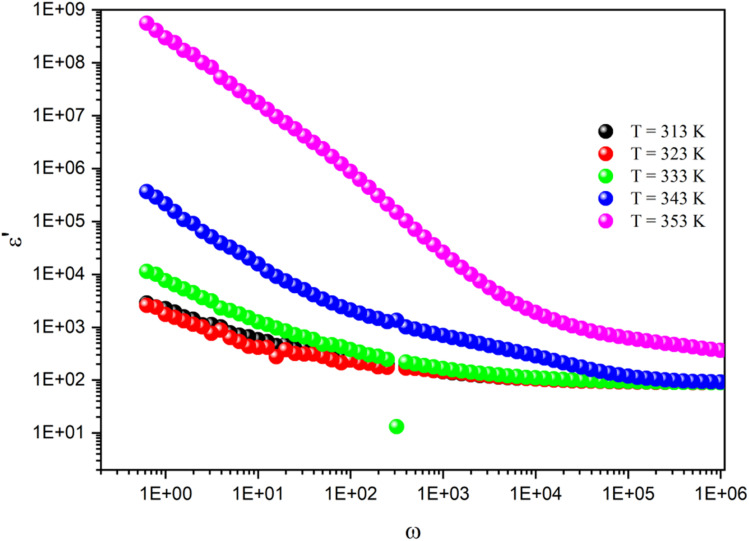
Frequency dependence of the dielectric constant at different temperatures.

Fig. 15 shows the frequency-dependences of the dielectric constant (*ε*′) at different temperatures. Two distinct regions could be observed in this variation. First, in the low-frequency region (*ω* < 10^3^ rad s^−1^), the material showed notably higher (*ε*′) values surpassing 10^9^ at 353 K, signifying their potential as efficient candidate for low-frequency energy storage.^71^ Compared to other organo–inorganic hybrid compounds, like [C_2_H_5_NH_3_]_2_CoCl_4_ (≈2 10^5^), [C_2_H_5_NH_3_]_2_ZnBr_4_ (≈4 10^6^)^51,72^ and [(C_6_H_5_N_2_)_2_ZnCl_4_] (10^3^–10^6^), the static permittivity of [(C_9_H_8_N)_2_(InCl_6_)·2(H_2_O)] was much higher. This could be attributed to the difference in the polarizability of the metal ions and the intermolecular interactions. These permittivity values have a significant impact on applications in electronic and optoelectronic devices, as high static permittivity can help improve the energy-conversion efficiency and the quality of light emission. Hybrid haloperovskites are widely used in these fields due to their exceptional optoelectronic properties. Second, as the frequency increased (*ω* > 10^3^ rad s^−1^), the *ε*′ motions showed a gradual decrease from 10^9^ to 10^2^ at 10^6^ rad s^−1^, which enhances the material's overall energy-storage efficiency.^73^ Furthermore, under fixed frequency, the dielectric constant increased with temperature, reflecting the thermal activation of charge carriers, which plays a crucial role in driving polarization mechanisms. These polarization phenomena fall into four key categories: ionic, electronic, orientational, and interfacial. Ionic and electronic polarizations, classified as deformational, result from shifts in the ion and electron positions in response to an electric field. Orientational and interfacial polarizations, categorized as relaxation components, involve the alignment of permanent dipoles and the interaction at the phase or material-electrode boundaries, respectively. These mechanisms collectively influence the material's dielectric behavior, which determines its energy-storage capabilities. The intricate interplay of temperature, frequency, and polarization types shapes the observed dielectric properties. Therefore, at low frequencies, the real part of the dielectric constant is shaped by orientational, interfacial polarizations and space charge effects, validating the non-Debye behavior of the material.^74^ However, in high-frequency scenarios, the electronic and ionic polarizations are more significant, and the electrons do not have sufficient energy for crossing the barriers, leading to lower dielectric constant values. The increase in temperature further enhances the real component of the dielectric constant, driven by thermally activated charge carriers and the behavior of electric dipoles, limiting the ability of electrons to cross barriers and thereby lowering polarization.^75^

## 5. Conclusion

In this work, we synthesized a new indium-based organic–inorganic hybrid compound formulated as [(C_9_H_8_N)_2_(InCl_6_)·2(H_2_O)] *via* slow evaporation at room temperature. SXRD analysis clarified that the crystal structure belonged to the centrosymmetric *P*1̄ space group. The consolidation and stabilization of the title compound was ensured through various types of hydrogen bonds. By utilizing infrared spectroscopy at room temperature, we were able to identify and confirm the different vibration modes and the composition of the crystal structure. The ultraviolet spectrum exhibited two absorption peaks at 264 and 365 nm, which were the main characteristics of its optical properties, with energy gaps of 3.96 and 4.74 eV, respectively. In fact, the thermal breakdown of the tested complex took place in three distinct stages, resulting in the decomposition of both the organic and inorganic entities. These stages led to a total mass loss of approximately (92.34%). Hirshfeld surface analysis was implemented to visualize and expound upon the disparities in the molecular surroundings between these two entities. Through the investigation of diverse intermolecular interactions using Hirshfeld surface analysis, it was discovered that the complex exhibited contributions from Cl⋯H/H⋯Cl and H⋯H contacts, which play a crucial role in determining the crystal structure of the new recently developed hybrid complex. The study of the compound's electrical conductivity indicated the semiconducting characteristics of the material. It was further shown that the conductivity response in alternating current aligned with the Jonscher power law at different temperatures. The decrease of *S* indicated that the transport mechanism in this material was governed by a correlated barrier hopping mechanism (CBH). The electrical data were evaluated using a modular approach that featured a unique relaxation time distribution and the Havriliak–Negami function. Finally, the material's high dielectric constant (10^9^) in comparison to other compounds suggests its potential for use in non-linear optoelectronic devices.

## Data availability

CDCC no. 2323035 contains the supplementary crystallographic data for the compound. This data can be obtained free of charge at https://www.ccdc.cam.ac.uk.

## Conflicts of interest

There are no conflicts to declare.

## Supplementary Material

RA-015-D5RA01127B-s001

RA-015-D5RA01127B-s002
