# Supplementary material for: Synthesis, crystal structure, vibrational study, optical characterization, Hirshfeld surface analysis and dielectric studies of a new indium-based hybrid material formulated as [(C9H8N)2(InCl6)·2(H2O)]
Source: RSC Adv. 2025 Apr 28;15(17):13628–42. doi: 10.1039/d5ra01127b (PMC12035700; doi:10.1039/d5ra01127b)
Supplement: RA-015-D5RA01127B-s001 [file RA-015-D5RA01127B-s001.pdf]

Supplementary Information (SI) for RSC Advances.
This journal is © The Royal Society of Chemistry 2025

Table S1 Fractional Atomic Coordinates (×10^4) and Equivalent Isotropic Displacement Parameters (Å^2×10^3). U_{eq} is defined as 1/3 of the trace of the orthogonalised U_{IJ} tensor.

Atom	*x*	*y*	*z*	U(eq)
In1	1	0	0.5	0.0410(2)
Cl3	0.8217(3)	0.3378(2)	0.47190(17)	0.066(5)
Cl2	0.8043(3)	-0.0344(3)	0.70412(16)	0.0657(5)
Cl1	0.7535(4)	-0.0195(4)	0.3761(3)	0.01101(9)
O1	0.3881(10)	0.2781(8)	0.4300(7)	0.084(18)
N1	0.2135(8)	0.4724(8)	0.2004(5)	0.0546(13)
C5	0.2850(9)	0.4298(9)	0.0817(6)	0.0448(14)
C4	0.2010(10)	0.5666(9)	-0.0249(6)	0.0518(15)
C6	0.4329(9)	0.2645(10)	0.642(7)	0.0534(16)
C1	0.769(10)	0.6260(10)	0.2233(8)	0.0618(18)
C7	0.4965(10)	0.2342(11)	-0.0586(7)	0.0608(18)
C9	0.2726(13)	0.5269(12)	-0.01479(7)	0.066(2)
C3	0.521(11)	0.7324(10)	0.0014(8)	0.0622(19)
C2	-0.076(11)	0.7632(11)	0.1208(9)	0.066(2)
C8	0.4160(13)	0.3661(12)	-0.1628(7)	0.068(2)

Table S2 Anisotropic Displacement Parameters (Å^2×10^3). The Anisotropic displacement factor exponent takes the form: $-2\pi^2[h^2a^{*2}U_{11}+2hka^*b^*U_{12}+\ldots]$.

Atom	U_{11}	U_{22}	U_{33}	U_{23}	U_{13}	U_{12}
In1	0.401(3)	0.373(3)	0.0355(3)	-0.005(2)	0.038(2)	-0.0082(2)
Cl3	0.953(13)	0.380(9)	0.525(10)	-0.0106(7)	-0.231(9)	-0.0045(8)
Cl2	0.741(11)	0.749(12)	0.429(9)	-0.0121(8)	0.184(8)	-0.0313(10)
Cl1	0.866(15)	0.102(2)	0.131(2)	-0.345(18)	0.112(16)	-0.0263(14)
O1	0.104(4)	0.59(3)	0.98(5)	-0.001(3)	-0.53(4)	-0.031(3)
N1	0.54(3)	0.66(4)	0.41(3)	-0.006(3)	-0.006(2)	-0.021(3)
C5	0.48(3)	0.56(4)	0.39(3)	-0.007(3)	-0.003(3)	-0.030(3)
C4	0.64(4)	0.59(4)	0.46(4)	-0.002(3)	-0.009(3)	-0.039(3)
C6	0.55(3)	0.61(4)	0.49(4)	-0.006(3)	-0.003(3)	-0.029(3)
C1	0.59(4)	0.67(5)	0.60(4)	-0.022(4)	0.002(3)	-0.022(4)
C7	0.59(4)	0.71(5)	0.64(5)	-0.025(4)	0.014(3)	-0.038(4)
C9	0.92(5)	0.83(6)	0.40(4)	-0.002(4)	-0.013(4)	-0.051(5)
C3	0.69(4)	0.51(4)	0.70(5)	0.004(4)	-0.020(4)	-0.029(4)
C2	0.63(4)	0.53(4)	0.83(6)	-0.21(4)	-0.006(4)	-0.018(3)
C8	0.92(5)	0.95(6)	0.43(4)	-0.25(4)	0.013(4)	-0.062(5)

Bond distances (Å)	Bond angles (°)	Bond angles (°)
In1 Cl3[1]= 2.5001(16)	Cl3[1] In1 Cl3= 180.0	C9 C4 C5 = 117.6 (7)
In1 Cl3= 2.5000(16)	Cl3[1] In1 Cl1 = 89.99 (8)	C3 C4 C5 = 118.4 (7)
In1Cl2[1]= 2.4559(16)	Cl3 In1 Cl1 = 90.01 (8)	C7 C6 C5 = 118.4 (7)
In1 Cl2 = 2.4559(16)	Cl3 Zn1 Cl1[1] = 89.99 (8)	N1 C1 C2 = 119.5 (7)
In1 Cl1= 2.521(3)	Cl3[1] In1 Cl1[1] = 90.01 (8)	C6 C7 C8 = 120.9(7)
In1 Cl1[1]= 2.521(3)	Cl2[1] In1 Cl3[1]= 89.60 (7)	C8 C9 C4 = 120.4 (7)
N1 C5= 1.368(8)	Cl2[1] In1 Cl3= 90.40 (7)	C2 C3 C4 = 122.1(7)
N1 C1= 1.304(9)	Cl2 In1 Cl2[1] = 180.0	C3 C2 C1 = 119.0(7)
C5 C4= 1.429(9)	Cl2[1] In1 Cl1[1]= 92.24(8)	C9 C8 C7 = 121.6(7)
C5 C6= 1.386(9)	Cl2 In1 Cl1= 92.24(8)	
C4 C9= 1.404 (10)	Cl2[1] In1 Cl1= 87.76 (8)	
C4 C3= 1.417(10)	Cl2 In1 Cl1[1]= 87.76 (8)	
C6 C7= 1.374(10)	Cl1 In1 Cl1[1]= 180.0	
C1 C2= 1.401(11)	C1 N1 C5= 125.4 (6)	
C7 C8= 1.387(11)	N1 C5 C4 = 116.4 (6)	
C8 C9 = 1.342 (12)	N1 C5 C6 = 122.5 (6)	
C3 C2 = 1.337 (11)	C6 C5 C4 = 121.1 (6)	

Table S3. Bond length (Å) and angles (°) of compound $[(C_9H_8N)_2(InCl_6)\cdot 2(H_2O)]$
